# The iron woman: a case report of contemporary cardiac support

**DOI:** 10.1093/ehjcr/ytaf371

**Published:** 2025-08-05

**Authors:** Jeremy A Brooksbank, Nikolaos Spilias, Carmela D Tan, Randall C Starling, Samir Kapadia

**Affiliations:** TriStar Centennial Medical Center, Advanced Heart Failure and Transplant Cardiology, 2400 Patterson St. Suite 502, Nashville, TN 37203, USA; University of Miami Miller School of Medicine, Interventional and Structural Cardiology, Miami, FL 33125, USA; Sydell and Arnold Miller Family Heart and Vascular Institute, Cleveland Clinic, Cleveland, OH 44195, USA; Sydell and Arnold Miller Family Heart and Vascular Institute, Cleveland Clinic, Cleveland, OH 44195, USA; Sydell and Arnold Miller Family Heart and Vascular Institute, Cleveland Clinic, Cleveland, OH 44195, USA

**Keywords:** Case report, Cardiomyopathy, Secondary mitral regurgitation, MitraClip, Carillon, LVAD, Transplantation

## Abstract

**Background:**

Modern cardiovascular practice utilizes various devices and procedures to treat functional and structural heart disease.

**Case summary:**

We report an interesting case of a female patient with heart failure with reduced ejection fraction (HFrEF) and severe mitral regurgitation who received the full spectrum of available device-based therapies including mitral transcatheter edge-to-edge repair, percutaneous mitral annuloplasty with the Carillon device, temporary mechanical circulatory support, durable left ventricular assist device, and eventually cardiac transplantation.

**Discussion:**

Management of HFrEF and functional mitral regurgitation is a common clinical issue for which numerous devices may be utilized to improve patient symptoms and outcomes. In this report, we review indications, supporting data, and anatomical relationships of some of these devices. We also highlight stepwise progression to advanced heart failure requiring temporary and durable mechanical circulatory support followed ultimately by heart transplantation.

Learning points

- This case highlights contemporary percutaneous therapies for dilated cardiomyopathy and secondary mitral regurgitation and presents a stepwise progression towards advanced heart failure therapies.
- By including cross-sectional gross pathology corresponding with radiographic and tomographic imaging, we draw attention to the anatomic relationship of various contemporary device-based therapies on the explanted heart.

## Introduction

Modern cardiovascular practice utilizes various devices and procedures to treat functional and structural heart disease. We report an interesting case of a female patient with advanced heart failure with reduced ejection fraction (HFrEF) and severe mitral regurgitation (MR) who received the full spectrum of available device-based therapies including mitral transcatheter edge-to-edge repair (TEER), percutaneous mitral annuloplasty with the Carillon^©^ device, temporary mechanical circulatory support (MCS), durable left ventricular assist device (LVAD), and eventually cardiac transplantation.

## Summary figure

**Figure:**
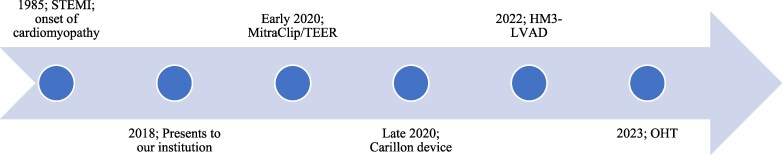


## Case presentation

A 68-year-old Caucasian woman with a history of ischaemic heart disease presented with one month of worsening New York Heart Association (NYHA) class III dyspnoea, orthopnoea, and palpitations. The patient had a past medical history of hypertension, premature ventricular contractions, and a large anteroseptal myocardial infarction (MI) at the age of 36 which was attributed to coronary vasospasm. Six months following MI, her left ventricular ejection fraction (EF) was 35%. She was followed intermittent by her primary care physician and maintained on medical therapy consisting of metoprolol succinate and losartan. She reported infrequent palpitations and dyspnoea on exertion, but had no other emergency department visits or hospitalizations in the 32 years since her MI.

**Figure 1:**
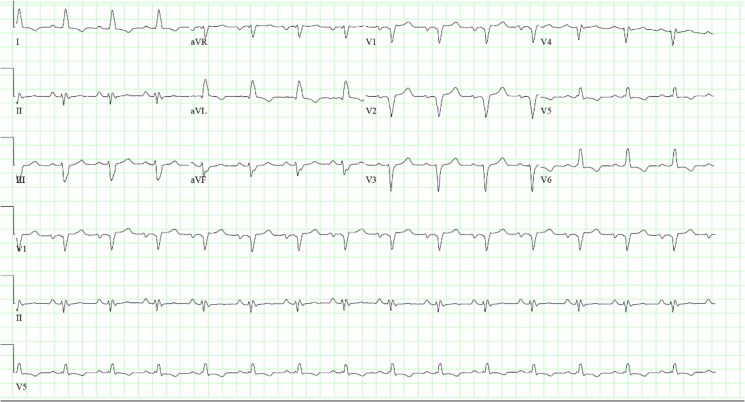
ECG upon presentation. Normal sinus rhythm with LBBB with QRS duration 122 ms.

On presentation to our facility, the patient had normal jugular venous pulsations, clear lungs, 1+ peripheral oedema, normal S1/S2 heart sounds, and a laterally displaced point of maximal intensity. ECG revealed sinus rhythm with left atrial enlargement and left bundle-branch block with QRS duration 122 ms (*Figure 1*). The patient underwent transthoracic echocardiography (TTE) which revealed reduced EF 24% with LV end-diastolic dimension (LVEDD) 7.0 cm and moderate-to-severe (3+) secondary MR. Left heart catheterization showed mild, non-obstructive coronary artery disease. Right heart catheterization revealed normal filling pressures [mean right atrial pressure 5 mmHg, pulmonary artery (PA) 42/12 (mean 22) mmHg, and mean pulmonary artery wedge pressure 10 mmHg] and severely reduced cardiac index (1.61 mL/min/m^2^). PA pulsatility index was normal at 6.0. She had additional optimization of guideline-directed medical therapy (GDMT) with maximally tolerated doses of metoprolol succinate (50 mg daily), transition to sacubitril–valsartan (24/26 mg twice daily), and initiation of spironolactone (25 mg daily) and dapagliflozin (10 mg daily). These medication changes led to improvement in symptoms, and she was discharged from the hospital with regular outpatient follow-up. Three months after medical optimization, TTE was repeated and showed interval improvement in EF to 45%.

The patient remained stable for 24 months prior to worsening symptoms prompting hospitalization. Transthoracic echocardiography at that time revealed reduction of EF to 20%, RV mildly dilated with normal systolic function, severe left atrial enlargement, and severe secondary MR due to apical tethering of the mitral leaflets in the setting of severe LV enlargement (LVEDD 7.8 cm). Transoesophageal echocardiogram showed structurally normal mitral leaflets with 4+ secondary MR (effective regurgitant orifice area 0.42 cm^2^) and holosystolic flow reversal in all pulmonary veins. After optimization of volume status with intravenous diuretics and appropriate adjustment of GDMT, a single-chamber implantable cardiac defibrillator (ICD) was placed for primary prevention of sudden cardiac death as EF remained <35%. Given these findings and after multidisciplinary heart team approach, she underwent mitral TEER with the deployment of 1 MitraClip (Abbott®) grasping the A2/P2 scallops of the mitral valve. The MR improved from 4+ to 1–2+ but worsened again a few months later leading to recurrent HF admissions. Transthoracic echocardiography showed a well-seated MitraClip device with moderate–severe MR and progressive mitral annular dilatation and LV enlargement. She then underwent compassionate percutaneous indirect mitral annuloplasty with placement of 80 mm Carillon mitral contour system (Cardiac Dimensions) in the coronary sinus, with the goal to reduce the dimensions of the mitral annulus and improve the LV geometry and MR. Echocardiographic images included in Supplementary material section, *Videos S1* and *S2*.

Despite temporary improvement in symptoms and MR following percutaneous mitral annuloplasty, she had progression of LV size and MR severity leading to stage D heart failure. Metabolic testing revealed adequate effort with respiratory exchange ratio (RER) 1.52 with peak oxygen uptake (pVO2) of 10.1 mL/kg/min (52% predicted) and minute-ventilation to carbon dioxide production ratio (VE/VCO2) slope 42. She was subsequently listed for cardiac transplantation or bridge-to-transplant LVAD. Prior to acceptable organ offer, she was admitted with cardiogenic shock and pulmonary artery catheter placement revealed RA 7 mmHg, pulmonary capillary wedge pressure (PCWP) 23 mmHg with pronounced v waves to >40 mmHg, and reduced CO (2.8) and CI (1.72).

**Figure 2:**
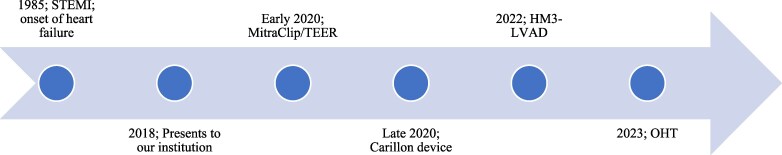
Summary figure with clinical timeline.

**Figure 3:**
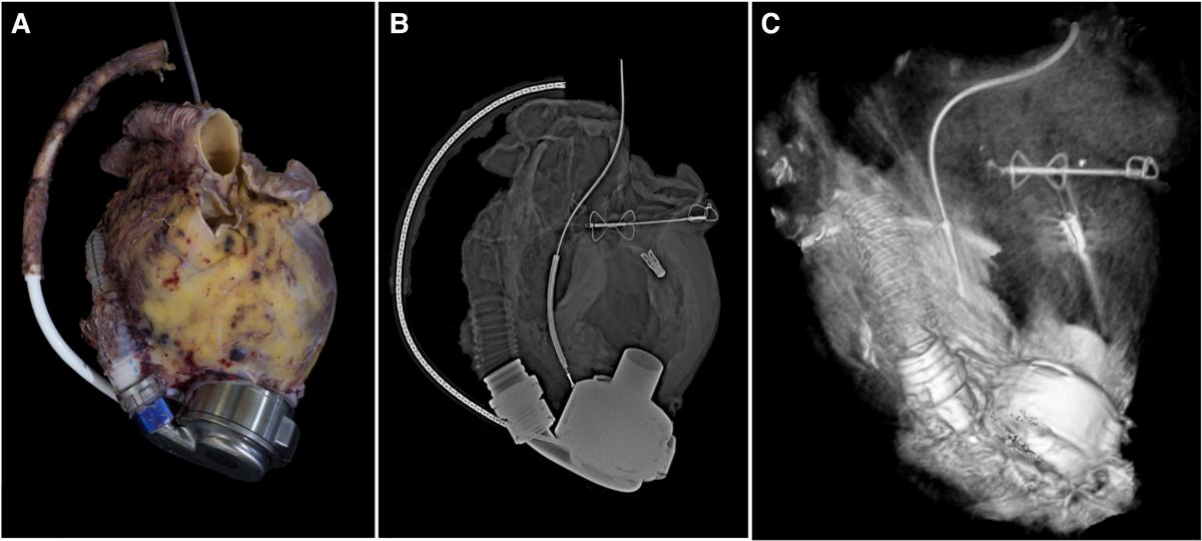
Gross anatomy of removed heart and associated supportive devices following heart transplantation. Image *A* is the gross pathologic images with corresponding radiographic (*B*) and CT reconstruction (*C*).

**Figure 4:**
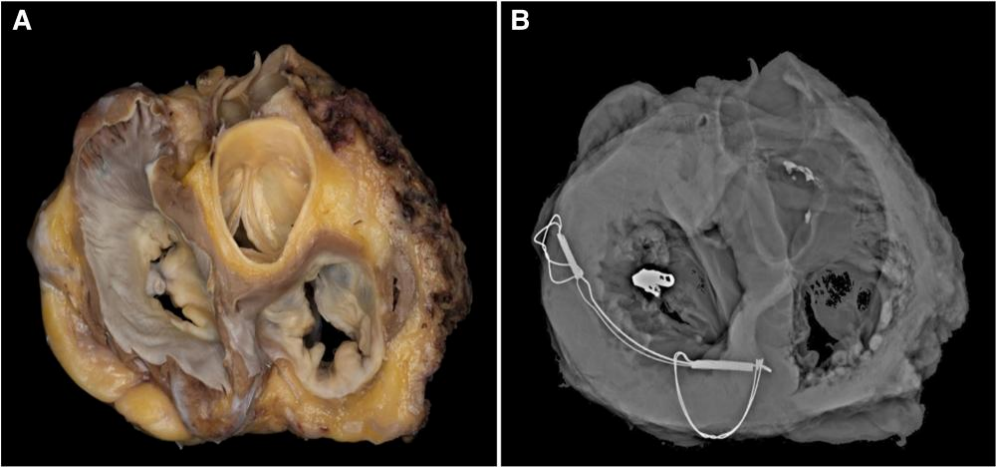
Short-axis view of gross anatomic specimen and corresponding radiographic image showing the three-dimensional relationship of cardiac anatomy and support devices.

She was stabilized with intra-aortic balloon pump (IABP) and subsequently underwent LVAD placement during admission. Thirteen months later, she underwent orthotopic heart transplantation (OHT). Please see *Figure 2* for clinical timeline. The explanted heart with its hardware underwent routine analysis and processing by the pathology department. Pathologic examination confirmed remote anteroseptal MI without significant coronary atherosclerosis. The mitral valve demonstrated one intact clip at A2/P2 scallops and a well-endothelialized Carillon device present in the coronary sinus. Pathologic specimens with corresponding gross, radiographic, and tomographic imaging are shown in *Figures 3* and *4*.

The patient recovered well after the OHT, with significant clinical improvement and improvement in quality of life. Routine surveillance endomyocardial biopsies showed subclinical 2R cellular rejection without any evidence of graft dysfunction at 1 week and 6 months post-transplant. The patient is having routine post-OHT follow-up.

## Discussion

This case highlights the contemporary device therapies for patients with severe cardiomyopathy/HFrEF and concomitant functional mitral regurgitation, which are provided within the context of a multidisciplinary heart team consisting of heart failure cardiologists, cardiac imagers, interventional cardiologists, and cardiac surgeons. It also provides a visual demonstration and understanding of the anatomic relationship between the different devices that can be used to treat advanced heart failure and prevent sudden cardiac death in the contemporary era, such as the ICD, the MitraClip device, the Carillon mitral contour system, and the LVAD.

Although the cornerstone of HFrEF management is optimization of GDMT, the response to GDMT remains variable, with progression of heart failure and increased morbidity and mortality in certain cases. With the exponential growth of structural heart interventions, minimally-invasive, device-based therapies have been developed and established for the treatment of HFrEF. Despite maximally tolerated GDMT, our patient continued to demonstrate clinical deterioration and progression of functional mitral regurgitation, leading to mitral TEER.

The 2022 AHA/ACC/HFSA guidelines denote a class IIa recommendation for transcatheter edge-to-edge MV repair (TEER) in patients with persistent NYHA class II–IV symptoms despite optimal GDMT and appropriate valvular anatomy with EF 20%–50%, LV end-systolic diameter (LVESD) ≤ 70 mm, and PA systolic pressure ≤ 70 mmHg.^1^

Despite initial improvement after TEER, continued LV dilation and MR progression may eventually ensue. The Carillon device (Cardiac Dimensions®) is a mitral annular reduction system which is implanted percutaneously in the coronary sinus with the goal to achieve reverse LV remodelling and MR reduction. The Carillon device was initially studied in the Titan I and Titan II trials to demonstrate device safety and efficacy.^2–4^ The randomized, sham-control, double-blinded REDUCE FMR Trial showed a reduction in MR and LV volumes with percutaneous mitral annuloplasty as assessed by echocardiography at 12 months.^5^ The ongoing, randomized, sham-controlled EMPOWER clinical trial will assess the effect of the Carillon mitral contour system in cardiovascular outcomes and LV remodelling in ∼300 patients with dilated cardiomyopathy and at least 1+ MR. The device was implanted via compassionate use in our patient and as a potential bridge to advanced heart failure therapies. Ultimately, and despite all the pharmacologic and device therapies, our patient progressed to stage D heart failure, requiring temporary MCS (IABP), LVAD placement, and eventually OHT.

The management of patients with HFrEF and secondary MR remains a challenge and requires the utilization of pharmacologic and device-based therapies within the context of the multidisciplinary heart team. A thorough understanding of the anatomy and mechanism of action of the available percutaneous and surgical devices is required for the timely selection of the appropriate therapy. Detailed examination of explanted hearts from patients undergoing heart transplantation may facilitate further understanding of function and anatomical relationships of current devices.

## Supplementary Material

ytaf371_Supplementary_Data

## Data Availability

Further echocardiography, CT, and gross pathology imaging are available from the senior author.

## Lead author biography

Dr Brooksbank earned his medical degree from University of Tennessee Health Science Center and completed his internship and residency in Internal Medicine from Duke University Medical Center in Durham, NC. After residency, Dr Brooksbank completed a Fellowship in Cardiovascular Medicine and Advanced Heart Failure and Transplant Cardiology (AHFTC) at Cleveland Clinic in Cleveland, OH. He works as an advanced heart failure cardiologist at Centennial Heart Cardiovascular Consultants in Nashville, TN.

## Supplementary material

**Consent:** Authors report receiving written informed consent from the patient prior to submission of this report, in accordance with COPE guidelines.

**Funding:** Authors report no industry partnerships or financial relationships relevant to this manuscript.
